# Infratentorial intracranial pressure monitoring: a hazardous option or an indispensable post-operative tool?

**DOI:** 10.1186/2045-8118-12-S1-P30

**Published:** 2015-09-18

**Authors:** Romain Manet, Laurent Gergele, Benjamin Pommier, Olivier Testa, Christophe Nuti, François Vassal, Jêrome Morel

**Affiliations:** 1 Department of Neurosurgery, University hospital of Saint-Etienne, France; 2 Department of Anesthesiology and Intensive Care, University hospital of Saint-Etienne, France

## Introduction

Due to its restricted size and the critical structures it contains, the posterior cranial fossa (PCF) is challenging for the neurosurgeon and each procedure exposes the patient to early and dramatic post-operative supratentorial (acute hydrocephalus) and/or infratentorial (brain swelling and/or hemorrhage) complications. Patient's monitoring after infratentorial surgery remains challenging. Indeed, supratentorial Intra Cerebral Pressure (ICPs) monitoring may not reflect infratentorial ICP (ICPi). Moreover, early post-operative CT scan is often difficult to interpret and occurrence of complications is lately suspected in the presence of clinical signs of brainstem impairment (bradycardia, pupillary dilation).

## Material and methods

Case report. Literature review.

## Results

We report the illustrative case of a 64 years old woman presenting severe bradycardia and bilateral mydriasis a few hours after a schedule infratentorial procedure. The CTscan reported cerebellar swelling and acute hydrocephalus. A second look surgery was performed for external ventricular drainage (EVD) and PCF decompression. ICP measured through EVD (ICPs) was low (5mmHg). Tactile estimation of intradural pressure was uncertain. Then a transdural intraparenchymal ICP transducer (ICPi) was inserted within the operated cerebellar hemisphere. During the initial post-operative course, monitoring showed significant difference between ICPi and ICPs during the first 24 hours (mean ICPi 17.2 mmHg +/- 2 vs mean ICPs 9.3 mmHg +/ -4.5) and during the 4 days after (mean ICPi 14.6 mmHg +/- 2.9 vs mean ICPs 7.8 mmHg +/- 3.1). The patient presented two episodes of severe bradycardia, with concomitant raise of ICPi above 20 mmHg (without significant change of ICPs and no over-drainage through EVD), treated successfully with osmotherapy. By means of optimisation of cerebral perfusion pressure (CPP) according to ICPi, patient was discharged of the Intensive Care Unit after 10days with moderate disability (GCS15, mRS4), and no complication of ICPi monitoring (no CSF leak).

## Conclusion

Due to “ancestral” fear of neurosurgeons to perform PCF drainage or ICP monitoring, in relations with former descriptions of major complications, ICPi monitoring remains a controversial and confidential practice. However, supratentorial ICP monitoring and hydrocephalus management can fail to prevent life threatening complications after infratentorial surgery or others PCF insults and ICPi monitoring should be evaluated to optimize post-operative management.

